# Association of Duodenal Atresia, Malrotation, and Atrial Septal Defect in a Down-Syndrome Patient

**Published:** 2016-04-24

**Authors:** R Angotti, F Molinaro, M Sica, F Mariscoli, E Bindi, O Mazzei, F Ferrara, M Messina

**Affiliations:** Division of Paediatric Surgery, Department of Medical, Surgical and Neurological Sciences, University of Siena, Siena, Italy

**Keywords:** Duodenal atresia, Down's syndrome, Malrotation, Atrial septal defect

## Abstract

Duodenal atresia is the frequent cause of neonatal intestinal obstruction. The association between duodenal atresia, intestinal malrotation, cardiac anomalies and Down syndrome is infrequently reported. We present a prenatally suspected case of duodenal atresia which was associated with malrotation and atrial septal defect in a patient of Down syndrome. Duodenotomy and resection of web was performed in addition to Ladd’s procedure. Postoperative course remained uneventful.

## CASE REPORT

A 1-day-old boy (weight 1.5kg), born through emergency caesarean section for maternal hypertension at 33+1, was evaluated for prenatal ultrasound findings of double bubble sign and polyhydramnios. Patient had typical features of Down syndrome and was already proven on prenatal testing. He was transferred to intensive care unit (ICU) for stabilization. Abdominal radiograph showed double-bubble sign. Nasogastric tube was placed. Echocardiography confirmed a prenatally suspected interatrial defect. Patient was hemodynamically stable. Surgery was performed on 3rd day of life in ICU (Fig. 1). At surgery, type-I duodenal atresia associated with malrotation was found. Duodenotomy and resection of diaphragm and Ladd’s procedure were performed. A trans-anastomotic tube was placed for 48 hours. Abdominal drain was placed for six days. Postoperative course was uneventful. Patient started orally on 8th postoperative day after upper gastrointestinal contrast study which was reported as normal. At last follow-up (6 months after surgery) the weight was 6.9 kg, and the weaning has been started without any problem. He is also under follow-up of cardiology team.

**Figure:**
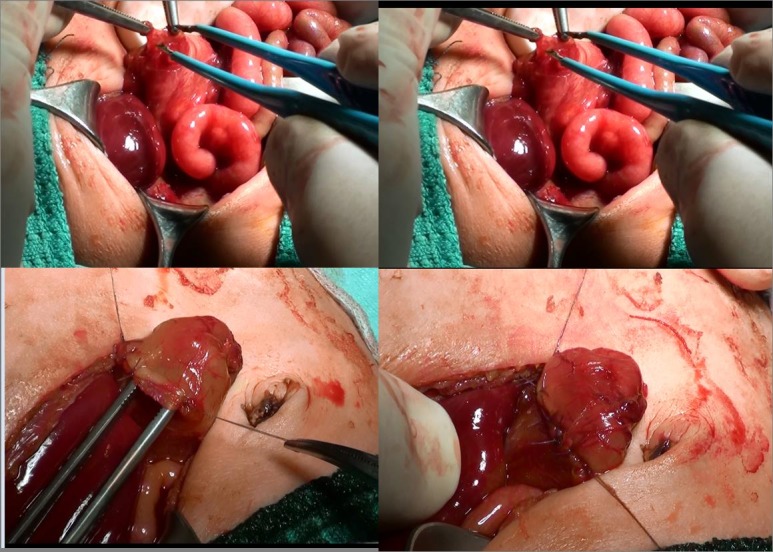
Figure 1:Intra-operative picture of duodenal web.

## DISCUSSION

Duodenal atresia is an isolated defect in 30-52% of infants. In more than 50% of patients it is associated with other congenital anomalies.[1-3] Down syndrome is present in approximately 20-30%, cardiac anomalies in about 20-25% of patients and malrotation in less than 20%. The presence/absence of associated anomalies has an important impact on prognosis. The reported survival rate of duodenal atresia is over 90% and the mortality is mainly due to associated complex cardiac anomalies. The presence of major cardiac malformation can delay the surgical repair. Presence of minor cardiac anomalies in index case did not increase risk during anaesthesia. The improved survival in duodenal atresia patients in the last two decades, is related to the prenatal diagnosis and provision of safe anaesthesia coupled with improved neonatal care during and after surgery.

## Footnotes

**Source of Support:** Nil

**Conflict of Interest:** None declared

